# Antimony thin films demonstrate programmable optical nonlinearity

**DOI:** 10.1126/sciadv.abd7097

**Published:** 2021-01-01

**Authors:** Zengguang Cheng, Tara Milne, Patrick Salter, Judy S. Kim, Samuel Humphrey, Martin Booth, Harish Bhaskaran

**Affiliations:** 1 State Key Laboratory of ASIC and System, School of Microelectronics, Fudan University, Shanghai 200433, China.; 2 Department of Materials, University of Oxford, Parks Road, Oxford OX1 3PH, UK.; 3 Department of Engineering Science, University of Oxford, Parks Road, Oxford OX1 3PJ, UK.; 4 Electron Physical Sciences Imaging Centre, Diamond Light Source Ltd., Didcot OX11 0DE, UK.; 5 Rosalind Franklin Institute, Harwell Campus, Didcot OX11 0FA, UK.

## Abstract

The use of metals of nanometer dimensions to enhance and manipulate light-matter interactions for emerging plasmonics-enabled nanophotonic and optoelectronic applications is an interesting yet not highly explored area of research beyond plasmonics. Even more importantly, the concept of an active metal that can undergo an optical nonvolatile transition has not been explored. Here, we demonstrate that antimony (Sb), a pure metal, is optically distinguishable between two programmable states as nanoscale thin films. We show that these states, corresponding to the crystalline and amorphous phases of the metal, are stable at room temperature. Crucially from an application standpoint, we demonstrate both its optoelectronic modulation capabilities and switching speed using single subpicosecond pulses. The simplicity of depositing a single metal portends its potential for use in any optoelectronic application where metallic conductors with an actively tunable state are important.

## Teaser

Antimony (Sb) acts as an ultrafast optical and optoelectronic nonlinear material at room temperature.

## INTRODUCTION

A large array of applications ranging from optical coatings (*1*), plasmonic antenna (*2*), metasurfaces (*3*), high-resolution imaging (*4*), biosensors (*5*) to integrated photodetectors and modulators (*6*, *7*) would greatly benefit from the development of active and nonlinear optical tunabilities in metallic states (*7*–*9*). Existing technologies to achieve active tunability by incorporating either tunable electro-optical materials (*10*), laser postprocessing (*11*), or electrolyte gating (*9*) are limited to low-speed, irreversible, or low-energy efficiency. Although the phase transition of metals between amorphous and crystalline structure has been studied since the 1960s (*12*–*14*), single-element metals have not been viewed as tunable optical materials. Even electronically, it was only recently that amorphous states of elemental metals have been obtained by nanosecond electrical pulse melt quenching (*15*–*17*).

Here, we report on antimony (Sb), which, when configured as a thin film of nanometric dimensions, behaves reliably as a tunable optical material. Such a functionality allows us to explore its use in a range of optical and optoelectronics applications as we demonstrate. The use of optical property contrast between two phases is not unknown in the context of a class of alloys known as phase-change materials (PCMs); it is no accident that those very properties of those materials have seen exploitation in photonic applications, including reflective nanodisplays (*18*), tunable emitters and absorbers (*19*, *20*), reconfigurable meta-photonics (*21*, *22*), and integrated phase-change photonics (*23*–*28*), accompanied by the development of specialized optical PCMs (*29*–*31*) and nanostructured optoelectronic devices (*32*, *33*). However, a common limitation with alloys is miniaturization, where maintaining compositional integrity is difficult at reduced dimensions.

We show that monoatomic metal materials with tunable nonvolatile optical properties could benefit photonic applications based on active metallic nanostructures and miniaturized metallic memories. Crystalline Sb (c-Sb) is a single-element metal, and its amorphous phase has been obtained by careful deposition of thin film (*14*, *34*) or electrical pulse switching (*15*, *17*) with notably decreased electrical conductivity working as a semiconductor. During the metal-insulator transition of Sb (*35*), a substantial change of the free carrier absorption will result in a remarkable contrast in its optical loss. Therefore, an optical property change of pure Sb could be expected during the phase transition, yet this has never been studied.

Here, we systematically studied the phase transition of ultrathin pure Sb in the optical domain using optical, electrical, and structural characterizations. We demonstrate that pure Sb is a promising tunable optical material with notable nonvolatile change in optical properties, especially the extinction ratio (*k*), between the amorphous and crystalline phases. We further demonstrate that pure Sb can be amorphized by a single-shot femtosecond pulsed laser with a tunable retention time of the switched amorphous phase. As we further show, this has substantial applications in reflective displays and potentially in future integrated photonics.

## RESULTS

### Material characterizations of ultrathin Sb films

**Fig. 1:**
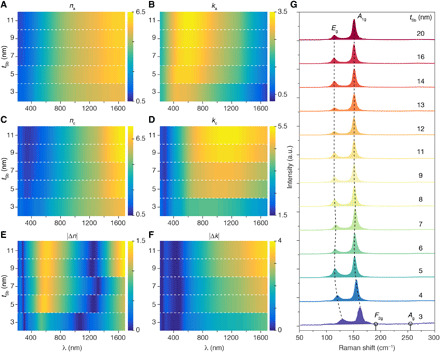
Thickness dependence on optical and structural properties of Sb. (**A** and **B**) The spectra (from ultraviolet to near infrared) of the refractive index *n*_a_ (A) and extinction ratio *k*_a_ (B) of thin-film Sb with different thicknesses (*t*_Sb_) as deposited on silicon wafers measured by spectroscopic ellipsometry. (**C** and **D**) The spectra of *n*_c_ (C) and *k*_c_ (D) of the same Sb samples in (A) and (B) after annealing on a hot plate (270°C for 10 min). (**E** and **F**) The absolute change of refractive index |Δ*n*| (|*n*_c_ − *n*_a_|) (E) and extinction ratio |Δ*k*| (|*k*_c_ − *k*_a_|) (F) of Sb upon annealing, calculated from (A) and (C) and (B) and (D), respectively. (**G**) Raman spectra of annealed Sb films with different *t*_Sb_. *E*_g_ and *A*_1g_ vibration modes are denoted by the dashed lines. Typical vibration modes *F*_2g_ and *A*_g_ of Sb_2_O_3_ are illustrated by the circles. The Raman spectrum intensity of 3-nm Sb (purple) has been enlarged by two times for clarification. a.u., arbitrary units.

First, we investigate the dependence of Sb thicknesses (*t*_Sb_) on optical constants, refractive index (*n*), and extinction ratio (*k*), as *n* and *k* are the key parameters for optical applications. Sb films with no capping layers were directly sputtered on silicon wafers and then characterized by ellipsometry measurements from which optical constants are determined (Fig. 1, A and B). For thin-film Sb (*t*_Sb_ ≤ 11 nm) as deposited, the dependence of refractive index *n*_a_ is weak in the ultraviolet and visible regimes (200 to 800 nm) with increasing *n*_a_ versus *t*_Sb_ in the infrared (Fig. 1A). Similarly, the extinction ratio *k*_a_ increases monotonically with *t*_Sb_ from visible to infrared yet on a larger scale (Fig. 1B). After annealing on a hot plate at 270°C for 10 min, the same samples were further investigated by ellipsometry with optical constants shown in Fig. 1 (C and D). Optical constants of ultrathin c-Sb (3 and 4 nm) do not follow the trend of thicker samples, with much smaller extinction ratio *k*_c_ values (Fig. 1D and fig. S1). Furthermore, when compared with amorphous Sb (a-Sb) samples, the refractive index change |Δ*n|* (|*n*_c_ − *n*_a_|) after crystallization is less than 1.5 (Fig. 1E), whereas the change in its extinction ratio |Δ*k*| (|*k*_c_ − *k*_a_|, Fig. 1F) is considerable with a maximum value over 3 in telecom wavelength bands (1.5 to 1.6 μm). The |Δ*k*| of Sb is much larger than that for GeSbTe (germanium-antimony-tellurium, or GST) and other PCMs (which is between 0.15 and 1.8 at 1.55 μm) (*36*). With increasing *t*_Sb_ (up to 20 nm), optical constants approach those of bulk Sb with less changes after annealing (fig. S1), which demonstrates that optical contrast of the phase transition can only be obtained from a thickness-confined thin film, less than 15 nm for the specific structure studied here. Despite the fact that the thickness-dependent optical property has been demonstrated in two-dimensional (2D) materials because of the quantum confinement and the interlayer coupling (*37*), this has not been widely reported in thin-film Sb.

In addition, Raman spectra of c-Sb samples have been investigated in Fig. 1G. Typical in-plane (*E*_g_) and out-of-plane (*A*_1g_) vibrational modes of Sb are denoted in the figure. For *t*_Sb_ larger than 9 nm, Raman peaks for *E*_g_ and *A*_1g_ are at ~114 and ~151 cm^−1^, consistent with that in bulk Sb (*38*). When *t*_Sb_ gradually decreases to 3 nm, both peaks for *E*_g_ and *A*_1g_ blue-shifted to larger wave numbers. Similar phenomena have been reported in 2D antimonene (*38*, *39*) relevant to local lattice contractions. It is worth noting that no antimony oxide (Sb_2_O_3_) Raman peaks at ~191 and ~255 cm^−1^ were observed in our samples, which confirms that the change in optical properties of Sb upon annealing is due to the phase change rather than from any oxidation. On the other hand, the Raman spectrum of Sb before annealing is insensitive to the thickness and shows an amorphous phase for all samples (fig. S2), indicating that all Sb films undergo the phase transition from amorphous to crystalline upon annealing. However, only thin-film Sb (*t*_Sb_ < 15 nm) has a remarkable change in optical and electrical properties. It has been suggested that local clusters of Sb (Sb_1_ or Sb_4_) are important to the electrical properties of ultrathin films and Raman spectra but have little effect on the electrical properties of thick Sb films (*14*). The amorphous phase of thick Sb (>15 nm) behaves more like a metallic glass (*16*) rather than a semiconducting material.

**Fig. 2:**
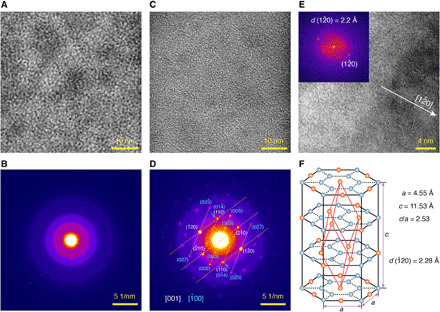
TEM characterization of Sb. (**A**) TEM image of a 5-nm-thick Sb layer as deposited has an amorphous structure. (**B**) Selected-area (344 nm diameter) electron diffraction (SAED) of the as-deposited Sb, corresponding to the sample in (A). (**C**) TEM image of the same Sb sample in (A) after thermal annealing. (**D**) SAED (200-nm-diameter region) of the annealed Sb sample in (B) has formed diffraction spots from crystalline planes. Two patterns, corresponding to the zone axis of [001] (white) and $\left[\overset{–}{1}00\right]$ (cyan), are overlapped. Miller indices of crystal planes with high symmetry are labeled. The arrows show merged diffraction spots coming from different planes. (**E**) TEM image of the annealed Sb with visible crystalline planes along the $\left[1\overset{–}{2}0\right]$ direction. Inset: Fourier transform of the whole image showing reflections $\left(1\overset{–}{2}0\right)$ with an interplane spacing (*d*) of 2.2 Å. (**F**) The hexagonal unit cell of the rhombohedral crystalline structure of Sb. The red lines show the primitive rhombohedral unit cell. *a* and *c* are the measured crystal constants in the hexagonal unit cell.

To identify the crystal structure of Sb before and after the thermal annealing, transmission electron microscopy (TEM) and selected-area electron diffraction (SAED) were implemented. Sb was sputtered as a 5-nm film on carbon films supported by copper grids. TEM of as-deposited Sb morphology is elucidated in Fig. 2A showing an amorphous disordered structure, which is confirmed by the diffusion halo pattern of SAED (Fig. 2B). After thermal annealing (270°C, 10 min), the morphology of Sb changed (Fig. 2C) with clear spot patterns in SAED (Fig. 2D), verifying its hexagonal crystalline structure. Two groups of diffraction patterns, corresponding to zone axis of [001] and $\left[\overset{–}{1}00\right]$, were observed in a 200-nm-diameter selected-area region. The TEM image in Fig. 2E shows crystalline planes perpendicular to the $\left[1\overset{–}{2}0\right]$ direction. The interplanar spacing was measured as 2.2 Å using the Fourier transform spots (inset of Fig. 2E). From the SAED pattern in Fig. 2D, we further calculated the crystalline constants *a* and *c* of the rhombohedral crystalline structure of Sb (Fig. 2F) as *a* = 4.55 Å and *c* = 11.53 Å (*c*/*a* = 2.53), consistent with bulk Sb (*a* = 4.31 Å, *c* = 11.27 Å, and *c*/*a* = 2.61) (*40*). Although the thickness of the Sb layer is only 5 nm here, its structure (orientation) is different from few-layer 2D van der Waals Sb (antimonene). The diffraction spots (006) and $\left(00\overset{–}{6}\right)$ corresponding to *c*-planes are clearly shown in Fig. 2D, which are typically missing in antimonene whose *c*-planes are parallel to substrates and perpendicular to the electron beam (*38*, *39*).

### Applications in strongly interfering optics

**Fig. 3:**
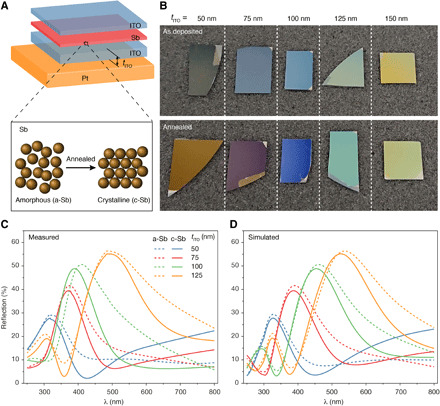
Switchable reflective stacks using ultrathin-film Sb. (**A**) Structure of reflective display based on phase-change Sb sandwiched between two ITO layers (ITO/Sb/ITO) on top of a Pt mirror. The phase-change Sb layer can be switched from amorphous to crystalline through thermal annealing. (**B**) Optical images show typical display samples with different thicknesses (*t*_ITO_) of the bottom ITO layer, while Sb and the top ITO are fixed at 5 and 15 nm, respectively. The samples have an amorphous (top row) and a crystalline (bottom row) Sb layer. (**C** and **D**) Measured (C) and simulated (D) reflection spectra of samples corresponding to (B). Each measured spectrum curve was normalized to the peak of the corresponding simulated curve.

Next, we explore how thin-film Sb responds within strongly reflecting thin-film structures. To do this, we demonstrate a reflective display structure (*18*) incorporating thin-film Sb PCMs. As shown in Fig. 3A, a thin-film Sb is sandwiched between two indium tin oxide (ITO) layers that have been deposited in sequence on a platinum (Pt) mirror. The thickness of Sb is fixed at 5 nm with a 15-nm top ITO capping layer. The reflective color of the sample is highly dependent on the thickness (*t*_ITO_) of the bottom ITO. We fabricated reflective display samples on silicon wafers with varying thicknesses of the bottom ITO: *t*_ITO_ = 50, 75, 100, 125, and 150 nm (Fig. 3B). The as-deposited thin Sb layer confined by ITO is in the amorphous phase (a-Sb). Reflective display samples incorporating a-Sb layers in the top panel of Fig. 3B show the reflective color changes from dark blue to bright yellow with increasing *t*_ITO_. To achieve fully c-Sb, we thermally annealed samples at 270°C for 5 to 10 min on a hot plate to produce reflective colors shown in the bottom panel of Fig. 3B. A notable color change was observed after the thermal annealing in all samples except for *t*_ITO_ = 150 nm. Both samples including a-Sb and c-Sb layers have been kept in atmosphere for 6 months without any color degradation. The color changes of these samples were further validated by the measured reflection spectra (Fig. 3C), where the maximum reflection peak is seen to shift red with the increase in *t*_ITO_ accompanied by a notable discrepancy of the spectra before and after annealing. This observation is consistent with the simulated reflective spectra in Fig. 3D, using a transfer matrix computational method (*15*) with optical constants for 5-nm Sb obtained by ellipsometry measurements (fig. S1). In addition, similar Sb stacks can be used in reconfigurable metasurfaces (*22*) and holographic displays (*41*) by optimizing the structure of the stack, with substantial applications in spatial light modulators and head-up displays for virtual and augmented reality.

### Optoelectronic modulation of Sb

**Fig. 4:**
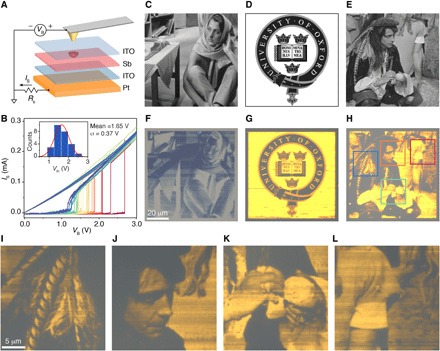
Electro-optical switching of Sb using conductive AFM. (**A**) Schematic of the electrical switching of Sb using CAFM. The Sb film is sandwiched between two ITO layers above a Pt mirror. The conductive probe of the CAFM is biased using a DC voltage (*V*_B_) while contacting or scanning the Sb sample. The Pt substrate is grounded via a resistor *R*_S_ (3 kilohms) to limit the current (*I*_S_) passing through the probe and the sample. (**B**) Static measurement of *I*_S_ while sweeping *V*_B_ on the probe that is in contact with different locations of the sample. Inset: Histogram distribution of the threshold voltage *V*_th_ of the switching during voltage sweeping. (**C** to **E**) Original grayscale (8-bit, 256 × 256) images used to modulate *V*_B_. (C) is from Marco Schmidt’s standard test images database, (D) is reproduced from the logo of the University of Oxford, and (E) is from the University of Southern California-Signal and Image Processing Institute (USC-SIPI) Image Database. (**F**) The optical image of the Sb sample switched by CAFM, corresponding to the image in (C). The Sb sample structure is 15-nm ITO/5-nm Sb/100-nm ITO/Pt (from top to bottom layer). (**G** and **H**) The optical image of the Sb sample switched by CAFM, corresponding to the images in (D) and (E), respectively. The Sb sample structure is 15-nm ITO/3-nm Sb/50-nm ITO/Pt. (**I** to **L**), Optical images show zoomed-in regions of the switched areas: blue (I), orange (J), green (K), and red (L) boxes in (H).

We then explore how thin-film Sb responds in the optoelectronic domain. To study this, we carry out electrical switching of the materials at nanoscale to investigate whether this results in optical contrast. Conductive atomic force microscopy (CAFM) is a versatile method used for nanoscale crystallization (*18*); we use CAFM to crystallize a-Sb with similar thin-film structures in Fig. 3. As shown in Fig. 4A, the Sb layer is encapsulated by the top and bottom ITO layers working as electrical contacts for Sb. The bottom ITO layer above the Pt layer is grounded through a protective resistor (*R*_S_). The conductive AFM tip is in contact with the top ITO layer with DC voltages applied, resembling the vertical structure of a standard phase-change memory cell. The current *I*_S_ passing vertically through Sb is monitored while the biased voltage *V*_B_ is varied. The current *I*_S_ is negligible at small *V*_B_ and rapidly increases to a high conductive state when *V*_B_ reaches a threshold voltage (*V*_th_), indicating a localized crystallization. We implemented the measurement over 20 different positions on the sample; this is shown in Fig. 4B, where the conductivity change is over two orders of magnitude during the switching consistent with previous studies on electrical switching of Sb (*14*, *15*, *17*), although with a wide distribution of *V*_th_ (inset of Fig. 4B). Next, grayscale images were patterned on Sb stacks by modulating *V*_B_ on the AFM tip while raster scanning the samples. For the stack of 15-nm ITO/5-nm Sb/100-nm ITO/Pt, the pixel color was switched from pale blue (a-Sb) to dark blue (c-Sb) by CAFM with the optical image taken in Fig. 4F, corresponding to the original picture in Fig. 4C. By reducing the thickness of Sb and further optimization of the stack (15-nm ITO/3-nm Sb/50-nm ITO/Pt), we have reached substantial improvement of the contrast of the switched images as shown in Fig. 4 (G and H), from original pictures in Fig. 4 (D and E, respectively). With this design, grayscale images have been perfectly replicated on the stack with a high resolution (<200 nm/pixel). The preservation of image detail is also very good (Fig. 4, I to L). Colors between a-Sb and c-Sb inferring intermediate phases have been achieved because of different bias voltages.

### Fast and reversible switching of Sb using femtosecond laser

**Fig. 5:**
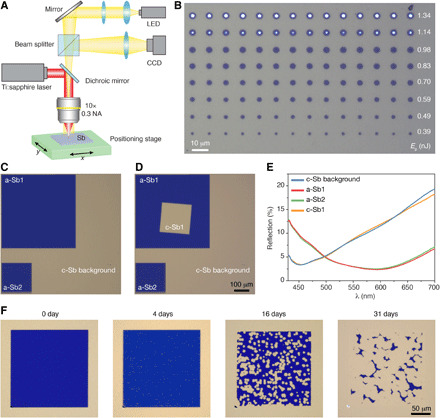
Ultrafast optical switching of Sb. (**A**) Schematic of optical switching of Sb using femtosecond laser. LED, light-emitting diode; CCD, charge-coupled device; NA, numerical aperture. (**B**) Optical image shows amorphized regions (blue disks) using single laser pulse (200 fs) with increasing energy *E*_p_ (from bottom to top). (**C**) Large area switching of the c-Sb sample (c-Sb background) through single femtosecond pulse (200 fs, *E*_p_ = 0.56 nJ) while raster scanning the sample (moving speed, 500 μm/s). a-Sb1 and a-Sb2 are switched areas with different sizes. (**D**) Recrystallization (c-Sb1) of the amorphized region a-Sb1 in (C) with multiple femtosecond pulses (200 fs, *E*_p_ = 29 pJ, 80 MHz) while translating the sample at 200 μm/s. (**E**) Reflection spectra of different locations of the sample in (D). (**F**) The stability of the amorphized region by femtosecond laser switching. Optical images show the amorphized region after different aging times in ambient conditions at RT. The Sb sample used is 15-nm ITO/3-nm Sb/50-nm ITO/Pt.

Last, we turn to studying both the reversibility of switching in these materials and their dynamic speed. In particular, for emerging applications in photonic computing, subnanosecond switching speeds are required, and faster speeds approaching picoseconds are highly desirable, which most PCMs are unable to reach. Earlier work on the amorphization of c-Sb using nanosecond electrical pulses at various ambient temperatures demonstrated that a faster process is necessary for the amorphization at room temperature (RT) or above (*15*). For this reason, we chose a femtosecond pulsed laser to optically switch Sb. The optical switching setup is illustrated in Fig. 5A; a regeneratively amplified Ti:sapphire femtosecond laser (λ = 790 nm, 1-kHz repetition rate, pulse of 200 fs) was focused on Sb samples through a 10× objective lens. Sb samples were mounted on a positioning stage for raster switching a large area. Similar to the electrical switching in Fig. 4, our sample is based on the ITO/Sb/ITO/Pt stack structure providing a good reflective color contrast that is readily observed using optical microscopes. An Sb stack sample (15-nm ITO/3-nm Sb/50-nm ITO/Pt) has been completely crystallized by thermal annealing. As shown in Fig. 5B, a single femtosecond laser pulse was used to amorphize the c-Sb stack sample with various pulse energies (*E*_p_). The switched region has a circular shape with a color change (to dark blue). The size of the switched region gradually increases with *E*_p_, until at very high power we ablate the entire stack (eventually exposing the underlying Pt at high *E*_p_). The switched regions were further characterized as a-Sb by Raman spectra (figs. S3 and S4), confirming that the color changes are a consequence of amorphization. Subsequently, large areas of a-Sb have been switched via the scanning of the sample stage while using single femtosecond pulse with a moderate energy (*E*_p_ = 0.56 nJ). Two typical amorphized regions (a-Sb1 and a-Sb2) are shown in Fig. 5C, with local reflection spectra measured in Fig. 5E suggesting robust and reproducible amorphization.

To demonstrate reversible switching, i.e., the recrystallization of switched a-Sb regions, a pulse train from the Ti:sapphire oscillator with 80-MHz repetition rate and substantially lower pulse energy (*E*_p_ = 29 pJ) was used as the laser source with the recrystallized region (c-Sb1) shown in Fig. 5D. The reflection spectra (Fig. 5E) and Raman spectra (fig. S5) of the recrystallized c-Sb1 are consistent with the background c-Sb. Since amorphized Sb has a strong tendency to recrystallize, we monitored the evolution of an optically switched a-Sb region at RT (~24°C), as shown in Fig. 5F and fig. S6. The switched a-Sb region was very stable over 36 hours; initial nucleation of the c-Sb after 4 days was observed and then followed by a gradual growth of nucleated regions. The whole recrystallization process for the amorphous region took more than 1 month. By slightly increasing the thickness of Sb to 5 nm, the initial nucleation in the optically switched a-Sb was decreased to ~24 hours at RT (fig. S7), which was further decreased to ~30 min at 40°C (fig. S8), resulting from the thickness and temperature dependence of the nucleation process. Moreover, the pulse energy used to amorphize the Sb also affects the retention time of a-Sb. While any pulse energy above a threshold can amorphize the Sb, a higher energy (below the damage threshold of the sample) typically gives less nucleation density accounting for a longer retention time for a-Sb (figs. S6 and S7). Notably, the retention time (at RT) of electrical switching of 3 and 5 nm Sb in (*6*) is ~50 hours and ~200 s, respectively, while our experiments using femtosecond optical pulses show a considerable improvement of the retention time above 30 days (Fig. 5F) and 18 days (fig. S7) for 3- and 5-nm Sb, respectively.

## DISCUSSION

Our experimental results have led to some very interesting observations, namely:

1) Compared to as-deposited thin-film Sb that is very stable (>6 months at RT), the optically amorphized a-Sb shows a much stronger tendency to recrystallize at RT. If the recrystallization of a-Sb is driven by the growth, it bypasses the nucleation (and speeds up the crystallization) that is required for the crystallization of as-deposited Sb films. On the other hand, if nucleation plays an important role, the optically switched a-Sb contains subcritical nuclei that facilitate the recrystallization (*42*), leading to a shorter retention time of a-Sb than the as-deposited Sb.

2) The strong optical contrast between a-Sb and c-Sb films can be attributed to different bonding mechanisms in the two phases (*43*–*45*). Sb with three *p*-electrons per atom and six nearest neighbors has been used as an isoelectronic model to discuss metavalent bonding (MVB; previously known as resonant bonding) in crystalline PCMs with aligned *p* orbitals; however, in an amorphous phase, MVB is broken or weakened by angular disorder resulting in a more isotropic bonding (covalent bonding for Sb). MVB gives smaller average bandgaps (*45*, *46*), resulting in larger refractive index observed in c-Sb than amorphous phases. Epitaxial growth of GeTe bilayers has shown that only above a critical thickness (four bilayers, ~1.4 nm) can the crystallization GeTe be reached (*46*), because the electron delocalization is greatly impaired in ultrathin films with the MVB weakened (*45*, *46*). It would be attractive to study optical properties and potential phase transitions of the ultrathin geometry of Sb as well, especially 2D antimonene, which can serve as an ideal platform to explore fundamental physics, such as thickness-dependent MVB in ultrathin systems.

3) It is known that the reduced glass-transition temperature *T*_rg_ = *T*_g_/*T*_m_ (*T*_g_ and *T*_m_ are glass transition and melting temperatures, respectively) is inversely related to the nucleation rate of PCMs (*13*, *42*). Therefore, we can calculate *T*_rg_ for Sb as 0.44, with *T*_g_ = 400 K (fig. S9) and *T*_m_ = 903.5 K (*47*). This value is smaller than for Ge_2_Sb_2_Te_5_ (*T*_rg_ = 0.47) and doped Sb (Ge_12_Sb_88_, *T*_rg_ = 0.53) (*48*), qualitatively indicating that Sb has a faster crystallization speed than conventional PCMs.

4) Thickness-dependent crystallization speed and temperature have been reported in other PCMs (*49*, *50*), which is explained by a qualitative model analyzing the energy barrier *E*_B_ for crystallization that determines the growth velocity of crystallites. The energy barrier *E*_B_ includes the crystalline-amorphous interfacial energy (*E*_ca_) and the crystalline-interface/surface energy (*E*_ci_). For an Sb film with thickness *t*, the initial growth of a crystalline cluster of radius *r* is dominated by *E*_ca_, given *r* < *t*/2. Once the size of the cluster surpasses *t* (*r* ≥ *t*/2), *E*_ci_, proportional to the crystalline-interface/surface area *S*_ci_ = π(*r*^2^ − *t*^2^/4), will contribute to *E*_B_. Therefore, for a given size of the crystalline cluster, thinner Sb has a larger *E*_ci_, leading to a stronger inhibition to the crystallization. On the other hand, randomly oriented a-Sb atoms activated by thermal energy will move to find a cluster structure with localized minimum energy for initial nucleation with a preference for internal rather than on the surface or interface nucleation. For ultrathin a-Sb, the ratio of surface or interface atoms to internal atoms is much larger than that in thick or bulk Sb. This results in ultrathin a-Sb taking a longer time to reach initial nucleation and subsequent crystallization. In addition, for thinner films when capped or sandwiched, related to a weakening of MVB, the viscosity of the amorphous phase increases to lower the Sb mobility and the crystallization rate (*45*). To fully understand the fundamental mechanism of the phase transition of Sb, especially the optical fast switching, advanced characterization, such as in situ TEM (*51*), femtosecond electron (*52*) and x-ray (*53*) diffractions, and phonon spectroscopy (*54*), accompanied by theoretical studies (*55*–*57*), is necessary and beyond the scope of this study.

5) Last, it is worth noting that Sb alloys such as GeSb have been intensively studied as fast crystallization PCMs for high-speed optical storage since the 1990s (*58*–*61*). In Sb-rich Ge_1–*x*_Sb*_x_* thin films (>20 nm; *x* > 0.85), the Ge content acts as a surface or interface in pure Sb to reduce the mobility of Sb atoms to reach a stable amorphous phase (*58*). The phase transition of Sb limits the ultimate speed of optical applications. Thus, similar to investigations on ultrafast switching of GeSb films (see Supplementary Text) (*59*–*61*), for future work, it is of great importance to study the time-resolved dynamics of optical property changes, via a pump-probe technique (*61*), under ultrafast optical pulse irradiation.

The potential applications of thin-film Sb in silicon photonics are many but require further investigation. Current integrated photonic memory elements mostly use GST and are based on the extinction ratio contrast between the amorphous and crystalline phases. Compared to GST, Sb has a larger extinction ratio for both a-Sb and c-Sb; however, the contrast is higher, indicating that photonic memory using Sb would have a smaller footprint that is crucial for cyclability and interfacing with electronics. Furthermore, our results indicate that Sb can be switched by a femtosecond laser pulse, portending subpicosecond time scales on integrated devices. In addition, the wide distribution of the retention time of a-Sb with tunable volatility can be used in photonic neuromorphic computing, to build photonic synapses (nonvolatile) and photonic neurons (volatile) by adjusting the thickness of the material (*62*).

In summary, we have explored the optical properties during the solid-state phase transition of a single metal Sb and find that its optical properties are unexpectedly tunable for a range of optical and optoelectronic applications requiring high-speed switching in a thin-film format. Optical constants (*n* and *k*) have a substantial contrast between the a-Sb and c-Sb when the thickness is less than ~15 nm. The thickness-dependent optical properties of Sb indicate that the interfaces of Sb have a substantial effect on the phase transition and optical properties. Electrical and optical methods, through CAFM and femtosecond laser, respectively, have been used to switch Sb, demonstrating high potential for versatile applications in nanophononics and optoelectronics. In future work, to optimize the switching speed, retention time, cyclability, and other performance metrics of Sb, the interfaces of Sb must be better understood. In addition, the volatility of the optically switched a-Sb can be modulated by the thickness, temperature, and the optical pulse energy for switching, indicating a potential material for synaptic and neuron functionalities, with promising applications in photonic neuromorphic computing, high-speed holographic and near-eye displays, and any other application that requires an actively tunable optical material with metallic properties.

## MATERIALS AND METHODS

### Film deposition

Thin films were deposited on silicon wafers (IDB Technologies) from commercial targets (99.99% pure, Testbourne) using radio frequency (RF) sputtering (Nordiko sputtering system). Sb films were sputtered at low RF power (30 W) and low-pressure (5 mtorr) Ar atmosphere with a deposition rate of 3.33 nm/min. Pt mirror was prepared by sputtering 100-nm Pt on silicon wafers at 50 W and 38 mtorr (8.6 nm/min), with a 5-nm Ta as the adhesion layer. ITO was sputtered at 30 W, 5 mTorr, and 2.28 nm/min.

### Material characterizations

Reflection spectrum in Fig. 3 was measured using ultraviolet–visible–near-infrared (UV-VIS-NIR) spectroscopy (Lambda 1050, PerkinElmer) fitted with a reflectance unit at an angle of incidence of 6°. Local reflection measurements in Fig. 5 were performed with a customized microscopy system, where a white light source was focused on the sample through a 20× objective lens (Thorlabs) with the reflection light collected by a single-mode fiber (M15L02-∅105 μm, Thorlabs) and detected by a portable spectrometer (OCEAN-FX-VIS-NIR, Ocean Optics). This customized microscopy system was also used to determine the crystallization temperature of Sb films on an in situ heating substrate (fig. S9). Ellipsometry measurement was implemented by a spectroscopic ellipsometer (RC2, J.A. Woollam) at three different incident angles. Refractive index and extinction ratio were obtained by fitting measurement results using built-in software, CompleteEASE (J. A. Woollam). Raman spectrum was measured by LabRAM ARAM1S (Horiba) using a 532-nm laser with a 50× objective lens, an 1800 grating, and a 25% filter. TEM characterization was taken by a LaB_6_ 200-kV transmission electron microscope (JEM-2100, JEOL) at the David Cockayne Centre for Electron Microscopy.

### Electrical and optical switching

An AFM (MFP-3D, Oxford Instruments Asylum Research) accompanied by a conductive diamond-coated tip (DDESP-FM-V2, Bruker) was used to electrically switch the Sb thin film sandwiched between ITO layers. For local switching of Sb, *V*_B_ was swept from 0 to 5 V and then back to 0 V while the current *I*_S_ passing through Sb was recorded. To switch a large area of Sb, the sample clamped on the piezo stage of the AFM was scanned at 1 kHz with a resolution of 512 points per line. The AFM tip was working in the contact mode with the biased voltage ranging from a minimum (0 V) to a maximum (6 to 8 V) value, corresponding to the grayscale value of the reference image used.

For optical switching, a regeneratively amplified Ti:sapphire laser (Solstice Ace, Spectra Physics) was the switching source, working at a wavelength λ of 790 nm and 1-kHz repetition rate with a pulse duration at the sample of ~200 fs. A single-pulse femtosecond laser with the energy *E*_p_ = 0.31 to 1.0 nJ was chosen to amorphize Sb samples. For the recrystallization of the a-Sb, 3000 consecutive pulses (at 1-kHz repetition rate) with an individual pulse energy of *E*_p_ = 0.16 nJ (total energy of 480 nJ per spot) were used, which required much slower translation of the sample (<0.3 μm/s). To save the total writing time, the laser source was switched to the oscillator that has a much higher repetition rate (80 MHz) but lower pulse energy (*E*_p_ = 29 pJ).

## Supplementary Material

http://advances.sciencemag.org/cgi/content/full/7/1/eabd7097/DC1

Adobe PDF - abd7097_SM.pdf

Antimony thin films demonstrate programmable optical nonlinearity

## SUPPLEMENTARY MATERIALS

Supplementary material for this article is available at http://advances.sciencemag.org/cgi/content/full/7/1/eabd7097/DC1
