# Supplementary material for: Antimony thin films demonstrate programmable optical nonlinearity
Source: Sci Adv. 2021 Jan 1;7(1):eabd7097. doi: 10.1126/sciadv.abd7097 (PMC7775754; doi:10.1126/sciadv.abd7097)
Supplement: http://advances.sciencemag.org/cgi/content/full/7/1/eabd7097/DC1 [file supp_7_1_eabd7097__index.html]

Science Advances | Science AdvancesAAASSearchScience AdvancesMenu

## Supplementary Materials

# Antimony thin films demonstrate programmable optical nonlinearity

Zengguang Cheng, Tara Milne, Patrick Salter, Judy S. Kim, Samuel Humphrey, Martin Booth, Harish Bhaskaran

Download Supplement

**This PDF file includes:**

- Supplementary Materials and Methods
- Supplementary Text
- Figs. S1 to S9

**Files in this Data Supplement:**

- Adobe PDF - abd7097\_SM.pdf
