# Supplementary material for: Antimony thin films demonstrate programmable optical nonlinearity
Source: Sci Adv. 2021 Jan 1;7(1):eabd7097. doi: 10.1126/sciadv.abd7097 (PMC7775754; doi:10.1126/sciadv.abd7097)
Supplement: http://advances.sciencemag.org/cgi/content/full/7/1/eabd7097/DC1 [file supp_7_1_eabd7097__1.pdf]

Science Advances
AAAS

advances.sciencemag.org/cgi/content/full/7/1/eabd7097/DC1

Supplementary Materials for

Antimony thin films demonstrate programmable optical nonlinearity

Zengguang Cheng*, Tara Milne, Patrick Salter, Judy S. Kim, Samuel Humphrey, Martin Booth, Harish Bhaskaran*

*Corresponding author. Email: zgcheng@fudan.edu.cn (Z.C.); harish.bhaskaran@materials.ox.ac.uk (H.B.)

Published 1 January 2021, *Sci. Adv.* **7**, eabd7097 (2021)
DOI: 10.1126/sciadv.abd7097

This PDF file includes:

Supplementary Materials and Methods
Supplementary Text
Figs. S1 to S9

Supplementary materials and methods

1. Film deposition

Silicon wafers (IDB Technologies Ltd., UK) were cleaned by solvents using acetone, isopropanol and DI water in sequence, followed by a baking process at 120 ºC on a hot plate for 5 mins. Afterwards, oxygen plasma was used to further remove contaminants and facilitate the surface adhesion of silicon wafer. Cleaned silicon wafers were taken as the substrate for material depositions. Thin films were deposited on silicon wafers from commercial targets (99.99% pure, Testbourne Ltd., UK) using RF sputtering (Nordiko sputtering system, Nordiko Technical Service Ltd., UK). **(1)** Sb films were sputtered on silicon wafers at low power (30 W) and low pressure (5 mTorr) Ar atmosphere with a deposition rate of 3.33 nm/min, without capping layers, used for ellipsometry measurements. **(2)** For ITO/Sb/ITO/Pt stack samples, 100 nm Pt was sputtered on silicon wafers at 50 W, 38 mTorr (8.6 nm/min) with a 5 nm Ta as the adhesion layer, followed by a deposition of ITO, Sb and ITO in sequence. ITO was sputter at 30 W, 5 mTorr, 2.28 nm/min and Sb was sputtered using the same condition as that for ellipsometry measurements.

Sb samples for TEM experiments were made as follow: **(1)** copper TEM grids with carbon support films (28~30 nm carbon film on 300 mesh grids copper, Agar Scientific Ltd., UK), used as received, were taken as the substrate for Sb film. **(2)** 5 nm Sb film was sputtered on copper TEM grids, same as the process mentioned above, hereafter, accompanied by a 5 nm SiO_2 film sputtered (80 W, 34.7 mTorr, 0.7 nm/min) on top as the capping layer.

2. Material characterizations

Reflection spectrum in Fig. 3 was measured using a UV-VIS-NIR spectroscopy (Lambda 1050, PerkinElmer Inc., USA) fitted with a reflectance unit at an angle of incidence of 6 degrees. Prior a real measurement, an aluminum mirror was used as a standard sample to take a background/reference scan. The mirror was then replaced by Sb stack samples to measure reflectance spectra that were calibrated with the reference scan. Local reflection measurements in Fig. 5 was performed with a customized microscopy system, where a white light source was focused on the sample through a 20× objective lens (Thorlabs, Inc., UK) with the reflection light collected by a single mode fiber (M15L02-∅105 μm, Thorlabs Inc., UK) and detected by a portable spectrometer (OCEAN-FX-VIS-NIR, Ocean Optics Inc., USA). The fiber core diameter (150 μm) determines the size of the sample area of which the reflection was collected from. This customized microscopy system was also employed to determine the crystallization temperature of Sb films on an in-situ heating substrate.

Ellipsometry measurement was implemented by a spectroscopic ellipsometer (RC2, J.A. Woollam Co., USA) at three different incident angles. Refractive index and extinction ratio were obtained by fitting measurement results using a built-in software CompleteEASE (J.A. Woollam Co.). The fitting model was based on a layer structure of Sb / 4.68 nm SiO_2 (native)/Si substrate. The thickness of the native oxide of silicon was obtained from reference measurements of silicon wafers using the same ellipsometer.

Raman spectrum was measured by LabRAM ARAM1S (Horiba, Ltd., Japan) using a 532 nm laser with a 50× objective lens, an 1800 grating and a 25% filter. **(1)** Unless stated otherwise, the accumulation time used was 1 s and 10 accumulations were taken for most

data points. **(2)** For amorphous Sb as-deposited samples of different thicknesses in Supplementary Fig. 2, an accumulation of 20 times was used to reduce the spectra noise. **(3)** Fewer accumulations (6~14) were used for optical switched squares of the 3 nm and 5 nm Sb samples in Supplementary Fig. 3 and 4, to eliminate the recrystallization by the Raman laser, whereas 5~7 accumulations were employed for optical switched spots of these samples due to the small switched areas being affected by the marginal amount of the drift of the Raman laser. **(4)** For switched areas of the 3 nm Sb in Supplementary Fig. 5, a 10% filter for the Raman laser was used. 2 accumulations each with 3 s of measurements were taken for optical amorphized squares while 1 accumulation (3 s) was used for the optical recrystallized region.

TEM characterization was taken by a LaB_6 200kV transmission electron microscope (JEM-2100, JEOL Ltd., Japan) at the David Cockayne Centre for Electron Microscopy. Images were collected on a Gatan Ultrascan camera (Gatan Inc., USA) and diffraction on an Orius camera (Gatan Inc.).

3. Sb switching

Thermal annealing: Sb samples were annealed on a conventional hot plate (Fisherbrand Isotemp, Fisher Scientific International Inc., USA) at 270 °C for 5~10 mins.

Electrical switching: An AFM (MFP-3D, Oxford Instruments Asylum Research, USA) accompanied by a conductive diamond coated tip (DDESP-FM-V2, Bruker, France) was used to electrically switch the Sb thin film sandwiched between ITO layers. As shown in Fig. 4A, the bottom mirror layer (Pt) was grounded through a protective resistor (3 kΩ) and the conductive tip was biased with a positive voltage V_B. For local switching of Sb, V_B

was swept from 0 V to 5 V then back to 0 V while the current I_S passing through Sb was recorded. To switch a large area of Sb, the sample clamped on the piezo stage of the AFM was scanned at 1 kHz with a resolution of 512 points/line. The AFM tip was working in the contact mode with the biased voltage ranging from a minimum (0 V) to maximum (6-8 V) value, corresponding to the grey scale value of the reference image used.

Fs laser switching: The schematic of the setup was shown in Fig. 5A. A regeneratively amplified Ti: Sapphire laser (Solstice Ace, Spectra Physics, USA) was the switching source, working at the wavelength λ of 790 nm and 1 kHz repetition rate with a pulse duration at the sample of ~200 fs. The output power of the linearly polarized pump laser was modulated using a rotatable half wave plate and a fixed Glan-Laser polarizer. The laser beam was focused on the Sb sample, mounted on a precision 3D positioning stage, through a 10 × objective lens (0.3NA, Zeiss, Germany) which gives a spot size of ~1.6 μm (0.6λ/NA). A white LED was used for illumination with the reflection image of the sample captured by a monochrome CCD camera. A dichroic mirror reflecting the laser beam but allowing the illumination light passing through, was employed to combine light beams to the objective. **(1)** A single pulse fs laser with the energy E_p = 0.31~1.0 nJ was chosen to amorphize Sb sample. To achieve large area switching to amorphous Sb, the laser was operated with a continuous pulse train emission while translating the sample relative to the fixed laser focus using the motorized positioning stage. The sample translation step between each incident pulse was 0.5~1 μm when moving at a speed (v) of 500~1000 μm/s, providing a uniform large area switching and an average pulse number per spot close to 1. **(2)** For the recrystallization of the amorphous Sb, 3000 consecutive pulses (at 1 kHz repetition rate) with individual pulse energy of E_p = 0.16 nJ (total energy of 480 nJ per spot)

were used, which required much slower translation of the sample (v < 0.3 μm/s). To save the total writing time, we subsequently switched the laser source to the oscillator that has a much higher repetition rate (80 MHz) but lower pulse energy. With this configuration, the sample stage could be moved at a much higher speed (v = 200~800 μm/s), while irradiating the sample with a continuous pulse train from the laser. The number of pulses per μm^2, N, can be estimated by considering the size of the laser spot, the repetition rate and the lateral spacing between each raster scan of the laser. For the recrystallization in Fig. 5D, N is estimated as 8×10^5 per μm^2 with the total energy (per μm^2) calculated as E_{sq} = 23.2 μJ/μm^2. ($N\times E_p$).

Notably, there is a large range in the total energy dose that may be used for the recrystallization process, which we did not yet fully characterize. When using the 80 MHz repetition rate oscillator for switching, energies E_{sq} in the range 10 to 55 μJ/μm^2 were all found to recrystallize Sb in Supplementary Fig. 5. However, we note that this dose is substantially more energy than is ultimately required, since we found also E_{sq} = 480 nJ/μm^2 was sufficient for recrystallization when using the 1 kHz repetition amplified laser source.

Supplementary text

Comparison of pure Sb with Sb-rich GeSb alloys

The role of Ge in GeSb. Sb alloys such as GeSb have been intensively studied as fast crystallization phase-change materials for high speed optical storage from 1990s to early 2000s (*58-61*). In Sb-rich $Ge_{1-x}Sb_x$ thin films (>20 nm; $x > 0.85$), the Sb content is dominant for fast crystallization times and strong optical contrast, while the Ge content is the ingredient to reduce the mobility of Sb atoms to reach a stable amorphous phase (*58*), in analogy to the role of surface or interface in pure Sb of this work.

Switching under ultrafast pulse irradiations. Reversible switching of GeSb films could only be achieved with a ps or fs laser pulse, whereas a fs laser pulse irradiation is a non-thermal process typically inducing a smaller crystallization and amorphization threshold fluences than a ps pulse (*59*), however, the transformation time in the amorphization process is longer for fs than ps pulses. This is because an ultrafast energy deposition under the fs pulse duration causes the recalescence effect which slows down the solidification in the amorphization process resulting in a longer transformation time (comparing with that under ps pulses) (*60*). It has been suggested that a thin-film system including a high thermal conductive substrate, a thin-film GeSb and a transparent atop layer could decrease the transformation time under fs pulses (*60*), which is in accordance with the thin-film Sb structure in this work suggesting a faster transformation time is possible for the case of pure Sb under a fs optical pulse.

Future work on Sb. The transformation time of the phase-transition of Sb is essential for the ultimate speed of optical applications, indeed for a future work it is of great

importance to study the time-resolved dynamics of optical properties changes, via a pump-probe technique (*61*), under ultrafast optical pulse irradiations.

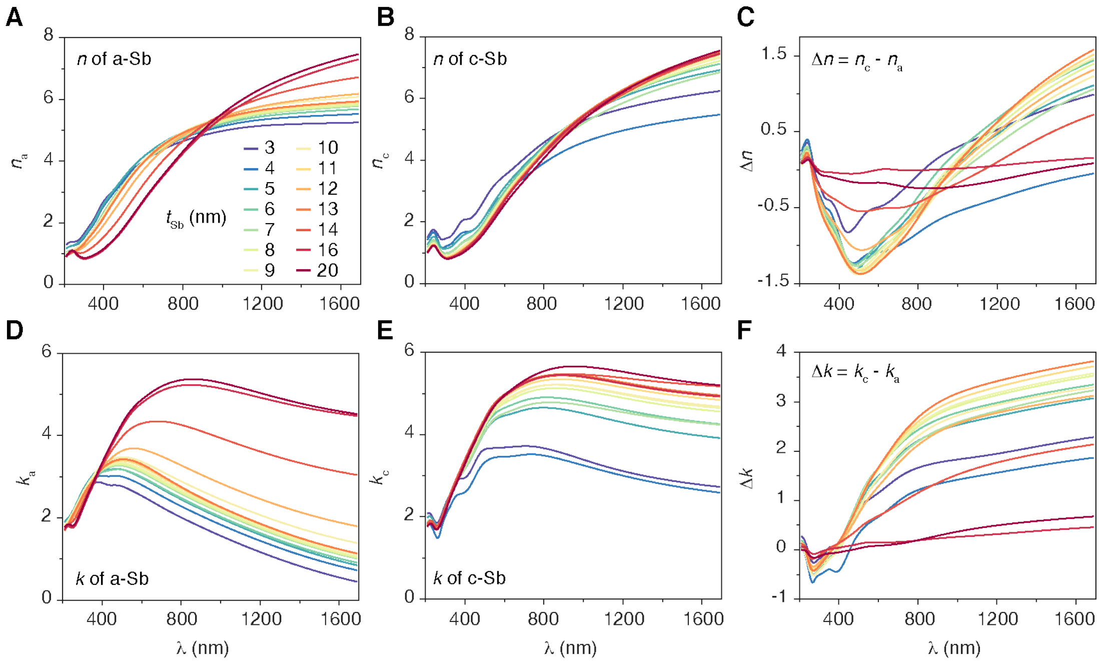

fig. S1 | Optical constants of Sb measured by spectroscopic ellipsometry.

(**A** and **D**), The spectra of refractive index n_a (A) and extinction ratio k_a (D)of a-Sb (as-deposited) with different thicknesses t_{Sb}. (**B** and **E**), The spectra of refractive index n_c (B) and extinction ratio k_c (E) of c-Sb (after annealing). (**C** and **F**) The spectra of refractive index change Δ*n* and extinction ratio change Δ*k* of Sb before and after annealing show thin film Sb with thickness less than 15 nm have substantial changes of optical constants while the changes for thicker Sb films (> 15 nm) are negligible.

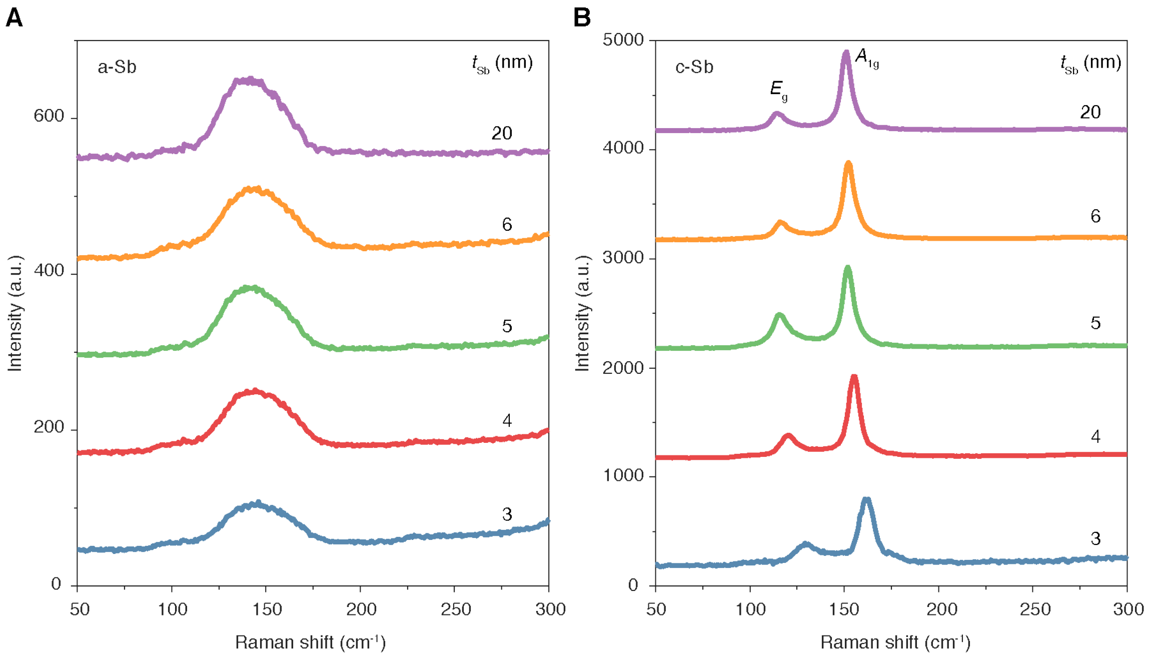

fig. S2 | Raman spectra of Sb films.

(**A** and **B**) Raman spectra of Sb with various thicknesses: t_{Sb} = 3, 4, 5, 6 and 20 nm, before (A) and after (B) annealing.

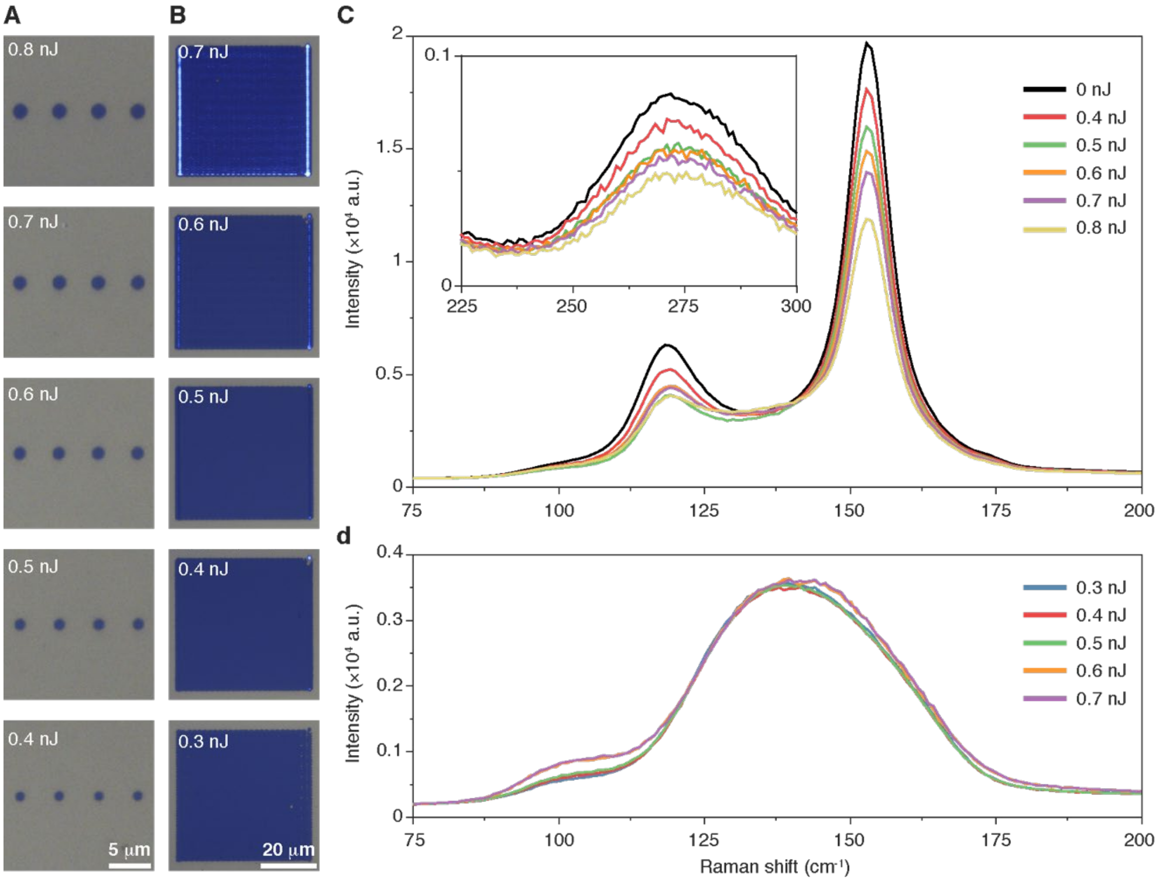

fig. S3 | Optical amorphization of 3 nm Sb using fs laser.

(**A** and **B**) Optical amorphization of a stack sample of 15nm ITO/3nm Sb/50nm ITO/Pt using fs laser: single spot switching (A) and raster scanning switching (moving speed 500 μm/s) (B). The energy used for switching is increased from bottom to top panels; (**C**) Raman spectra measured around amorphized spots in (A). As the sizes of switched a-Sb spots are smaller than the beam spot of the Raman laser, the spectra measured are mixture of a-Sb and c-Sb. With the increasing energy of the fs pulse, the sizes of a-Sb spots increase with the decreasing of the Raman peaks corresponding to c-Sb; (**D**) Raman spectra measured inside switched blocks in (B).

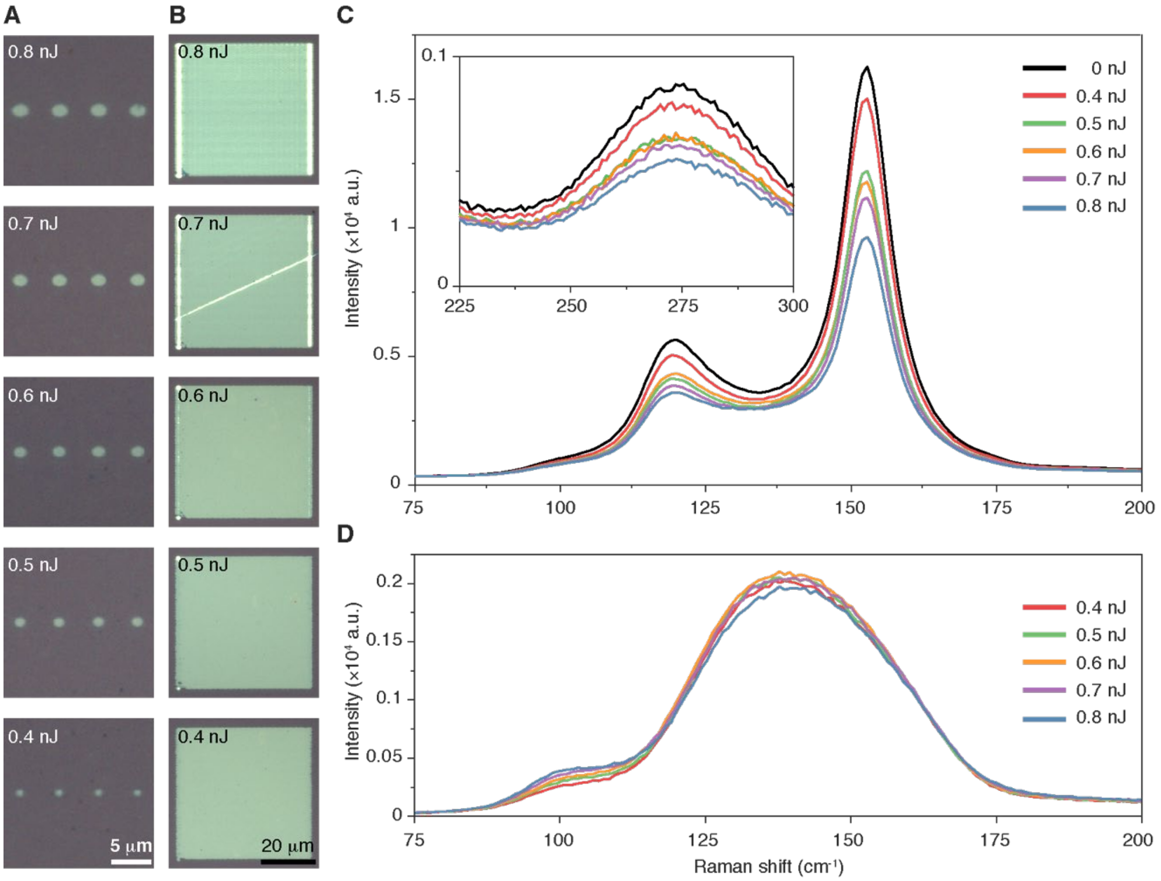

fig. S4 | Optical amorphization of 5 nm Sb using fs laser.

(**A** and **B**) Optical amorphization of a stack sample of 15nm ITO/5nm Sb/100nm ITO/Pt using fs laser: single spot switching (A) and raster scanning switching (moving speed 500 μm/s) (B). The energy used for switching is increased from bottom to top panels; (**C**) Raman spectra measured around amorphized spots in (A). The spectra measured are mixture of a-Sb and c-Sb. With the increasing energy of the fs pulse, the sizes of a-Sb spots increase with the decreasing of the Raman peaks corresponding to c-Sb; (**D**) Raman spectra measured inside switched blocks in (B).

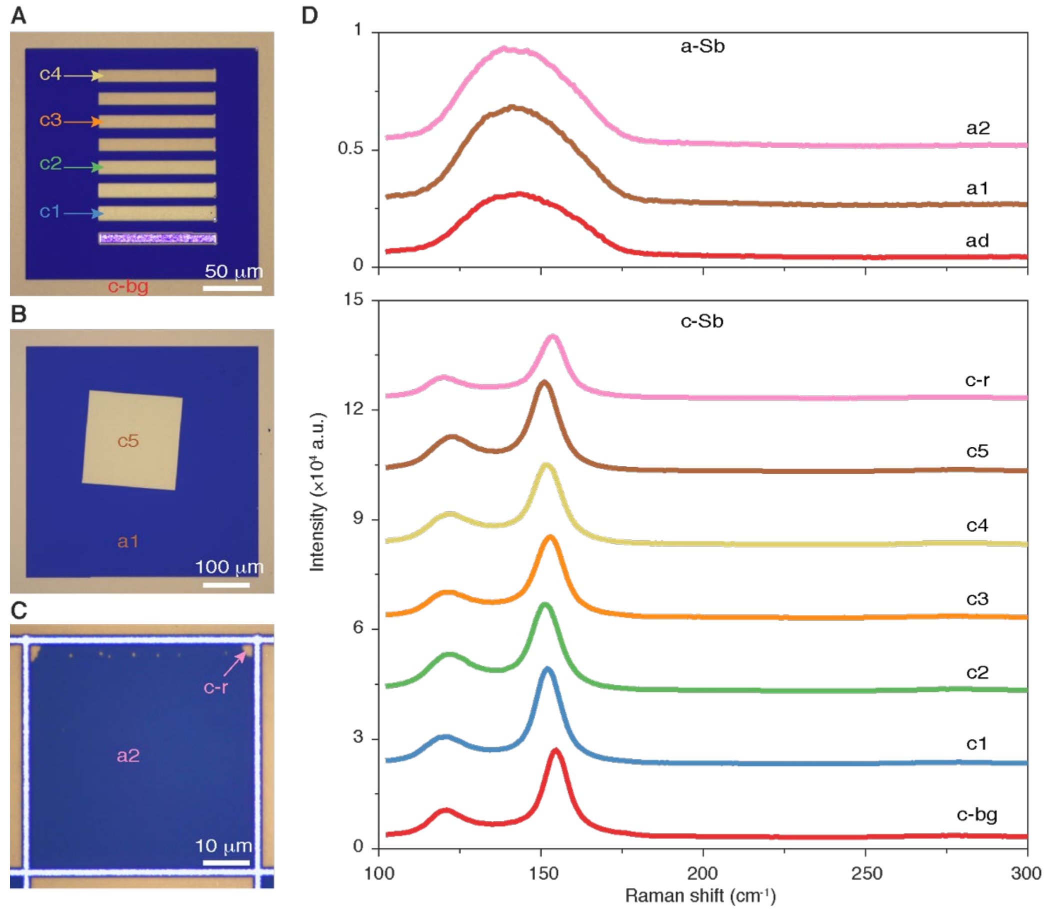

fig. S5 | Reversible switching of Sb using fs laser.

(**A** and **B**), Blue rectangular block is amorphized using single pulse fs laser while raster scanning a c-Sb sample (the sample was moved at 500 μm/s during the writing). Yellow regions inside blue blocks are recrystallized with multiple fs pulses (80 MHz). c-bg, crystalline Sb (c-Sb) sample prepared by thermal annealing; a1 and a2, amophozied Sb by optical switching; c1, c2, c3, c4 and c5, recrystallized regions using multiple fs pulses with each energy of: 68.75 pJ, 26.25 pJ , 12.5 pJ , 12.5 pJ and 29.13 pJ; The sample was moved at the speed of 100 μm/s for c3, 200 μm/s for c1, c2 and c5, 800 μm/s for c4. (**C**), Self-crystallization (c-r) of optical switched amorphous region (a2) after 48 hours. (**D**) Raman spectra of different locations in (A), (B) and (C). The sample stack is 15nm ITO/3nm Sb/50nm ITO/Pt. ad, amorphous Sb (a-Sb) sample as deposited. The sample is kept at RT.

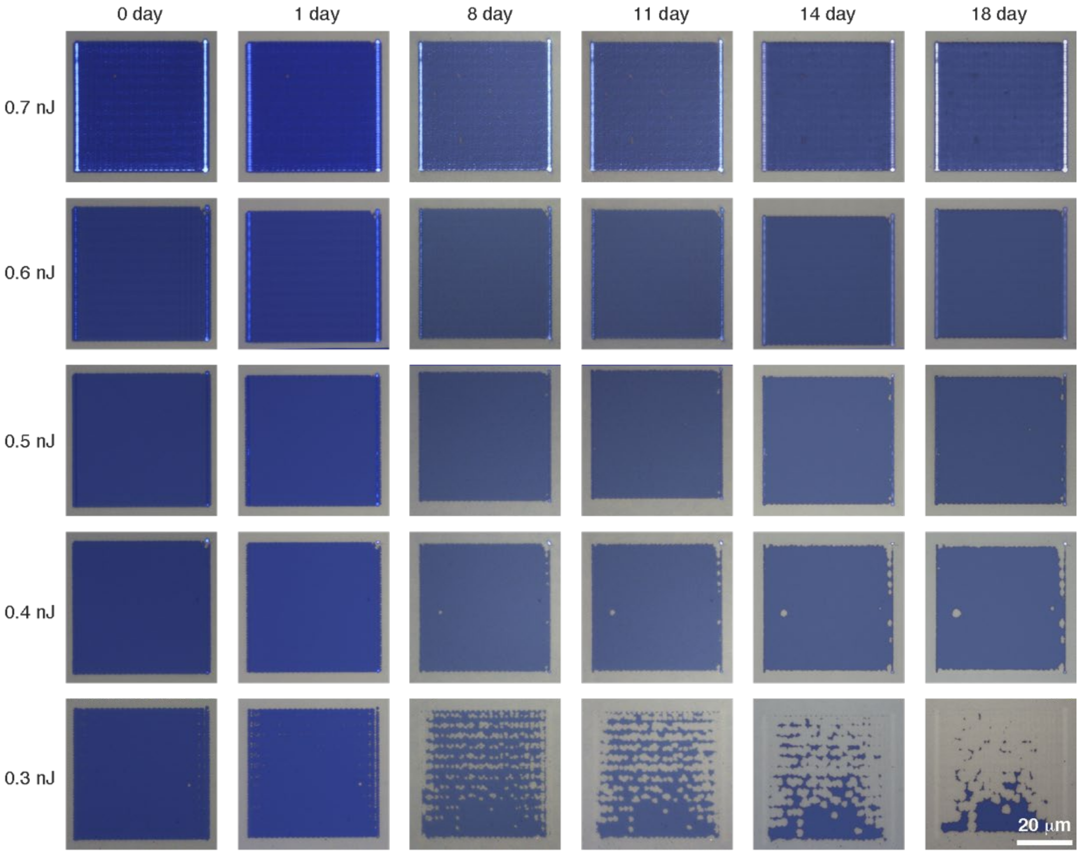

fig. S6 | The retention of a-Sb (3 nm) upon different optical switching energies.
The Sb stack of 15nm ITO/3nm Sb/50nm ITO/Pt is amorphized by fs pulses with different energies (from bottom to top). Optical images of switched regions are taken at different time (from left to right) after switching. The sample is kept at RT.

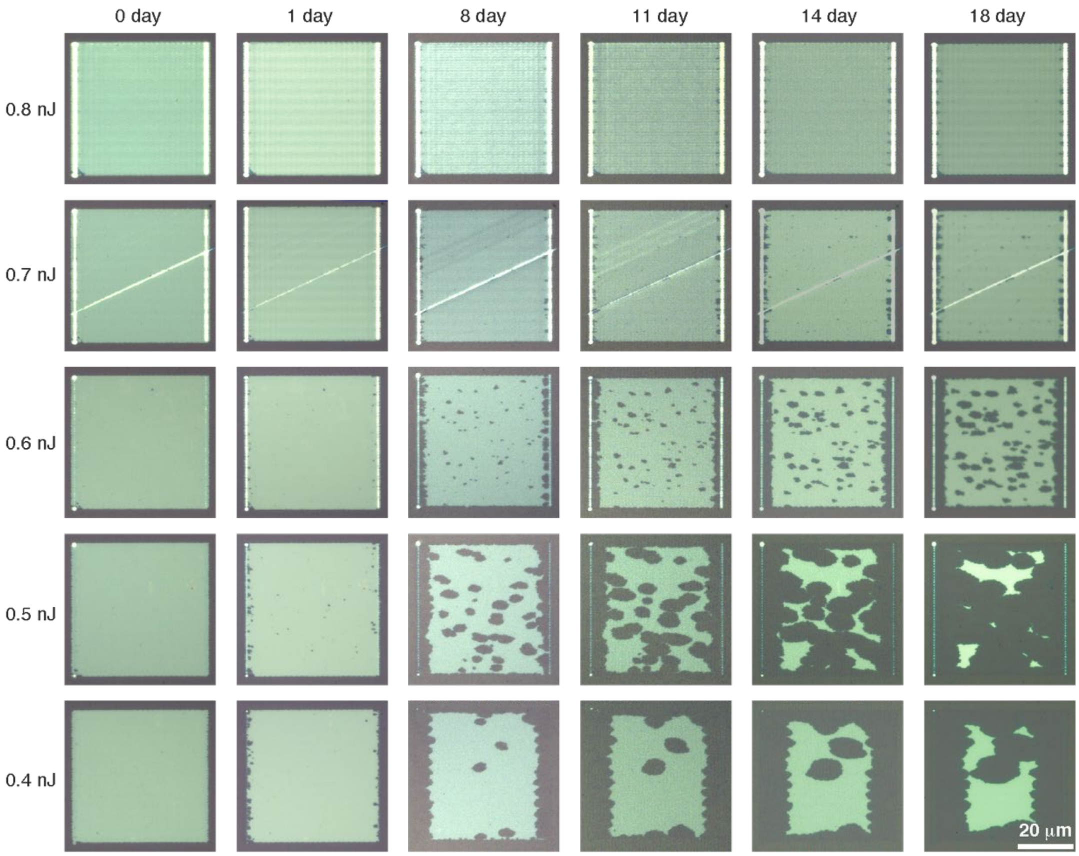

fig. S7 | The retention of a-Sb (5 nm) upon different optical switching energies.
The Sb stack of 15nm ITO/5nm Sb/100nm ITO/Pt is amorphized by fs pulses with different energies (from bottom to top). Optical images of switched regions are taken at different time (from left to right) after switching. The sample is kept at RT.

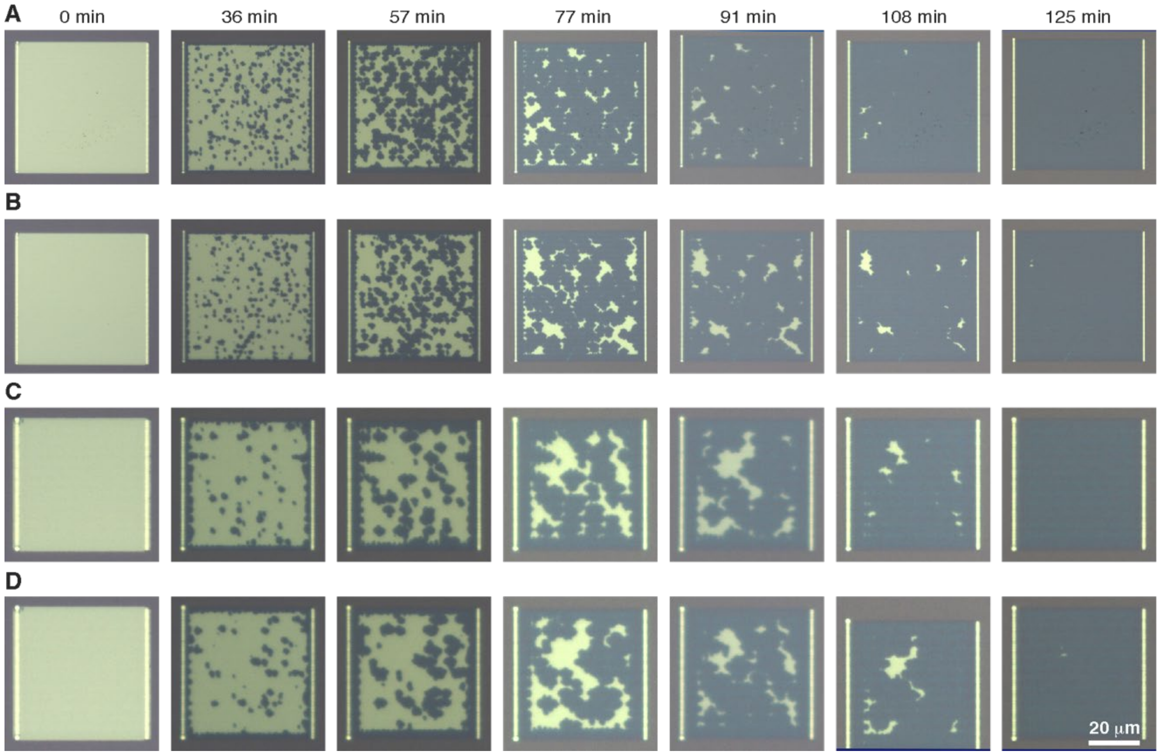

fig. S8 | The retention of a-Sb (5 nm) kept at 40 °C.

The Sb stack of 15nm ITO/5nm Sb/100nm ITO/Pt is amorphized by fs pulses. Optical images of switched regions are taken at different time (from left to right) after switching. The sample is kept at 40 °C on a heating plate.

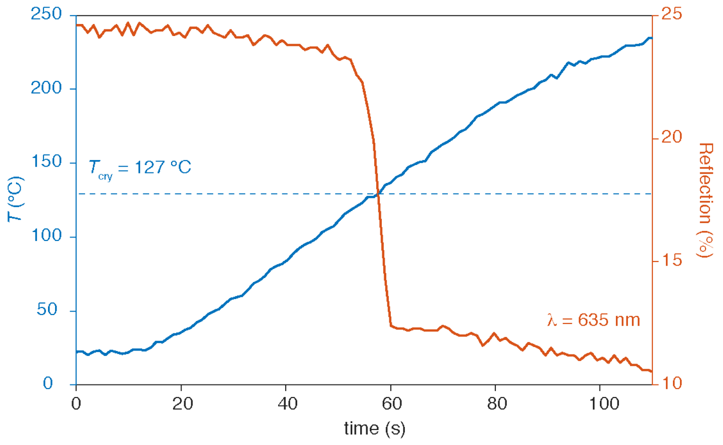

fig. S9 | Measurement of crystallization temperature (T_{cry}) of Sb film.
5 nm Sb film on silicon substrate was slowly heated whilst a continuous wave laser (λ = 635 nm) measured the reflection from the film. When temperature reaches T_{cry}, the Sb film switches to a crystalline phase with a rapidly dropping of the reflection. T_{cry} = 127 °C is determined by the highest first order derivative of the reflection trace. It has been studied that the glass transition temperature T_g is close to T_{cry} (*42*).
